# Online Movement Correction in Response to the Unexpectedly Perturbed Initial or Final Action Goals: An ERP and sLORETA Study

**DOI:** 10.3390/brainsci11050641

**Published:** 2021-05-15

**Authors:** Lin Yu, Thomas Schack, Dirk Koester

**Affiliations:** 1 Neurocognition and Action-Biomechanics Research Group, Faculty of Psychology and Sports Science, Bielefeld University, 33501 Bielefeld, Germany; thomas.schack@uni-bielefeld.de (T.S.); dirk.koester@businessschool-berlin.de (D.K.); 2 Center of Excellence-Cognitive Interaction Technology (CITEC), Bielefeld University, 33619 Bielefeld, Germany; 3 Sport Psychology, BSP Business School Berlin, 12247 Berlin, Germany

**Keywords:** movement execution, online correction, action goal, motor re-planning, grasping, middle frontal gyrus (MFG)

## Abstract

In this experiment, we explored how unexpected perturbations in the initial (grip posture) and the final action goals (target position) influence movement execution and the neural mechanisms underlying the movement corrections. Participants were instructed to grasp a handle and rotate it to a target position according to a given visual cue. After participants started their movements, a secondary cue was triggered, which indicated whether the initial or final goals had changed (or not) while the electroencephalogram (EEG) was recorded. The results showed that the perturbed initial goals significantly slowed down the reaching action, compared to the perturbed final goals. In the event-related potentials (ERPs), a larger anterior P3 and a larger central-distributed late positivity (600–700 ms) time-locked to the perturbations were found for the initial than for the final goal perturbations. Source analyses found stronger left middle frontal gyrus (MFG) activations for the perturbed initial goals than for the perturbed final goals in the P3 time window. These findings suggest that perturbations in the initial goals have stronger interferences with the execution of grasp-to-rotate movements than perturbations in the final goals. The interferences seem to be derived from both inappropriate action inhibitions and new action implementations during the movement correction.

## 1. Introduction

In everyday life, manual actions such as grasping can be produced effortlessly even if the external environment is changed unexpectedly. Whenever an ongoing prehensile action is no longer suitable for the current situation, individuals constantly correct it to comply with new task demands. The movement correction reflects a compensatory motor control mechanism, which comprises a series of efficient cognitive processes, such as a rapid online comparison between the contextual and motoric information (incompatibility detection), a suppression of prepared but inappropriate actions (issued action inhibition), and then the initialization of appropriate actions (novel action implementation) [1,2,3,4]. These processes take place and can be completed in a concise period after the change happens, even if the movements are relatively complex [5,6,7]. With the help of these efficient processes, movement correction facilitates humans to survive potential dangers and also supports individuals to interact adaptively with the dynamic world.

It has been implicated that the frontoparietal network is recruited in movement corrections; the network involves the pre-supplementary motor area (pre-SMA), the supplementary motor area (SMA), the anterior cingulate cortex (ACC), the inferior frontal gyrus (IFG), the premotor cortex (PMC), the intraparietal sulcus (IPS), the superior parietal lobule (SPL), and the supramarginal gyrus (SMG) [1,8,9,10,11,12,13,14,15,16,17]. Among them, the prefrontal cortical areas, such as the pre-SMA, ACC, and IFG, have been associated with detecting the incompatibilities between the contextual and motoric information as well as inhibiting ongoing but inappropriate actions [9,18,19]. Previous event-related potential (ERP) studies [20,21] reported that mid-frontal N2 and P3 components were elicited when a pre-planned response was successfully corrected, and the following source analyses found that these components were mainly derived from the ACC and pre-SMA. Transcranial magnetic stimulation (TMS) studies [15,22,23] also reported that virtual lesions in the IFG or pre-SMA impaired the performance of individuals’ movement adjustments. The ventral portion of the PMC (PMv) was also involved in the inhibition of a pre-planned action during movement corrections [14]. Meanwhile, the dorsal portion of the PMC (PMd) was employed for updating a pre-planned movement, which is not merely involved in inhibiting the issued inappropriate actions, but also initiating the appropriate actions [11,16,24]. The parietal cortical areas, such as the IPS and SPL, were relevant to planning and controlling goal-directed reaching or grasping movements [13,25]. In the correction of manual actions, the anterior portion of IPS (aIPS) is responsible for updating goal-related information and implementing new actions [1,8,26], whereas the SPL is mainly engaged in the real-time adjustments of the movement [8,27].

In previous studies, researchers have often focused on the movement corrections compensating for the perturbations in the recruitment of movement effectors (such as from one finger to another finger) [11,16,20,21,28,29] or the changes in physical properties (shape, size, orientation) of the target object [1,8,30,31,32,33,34]. For example, in a reach-to-grasp task [8], participants were instructed to pincer-grasp a wooden cuboid on the narrow side (1 cm) if it was horizontally oriented, or on the wide side (5 cm) if it was vertically oriented. The cuboid was always horizontally oriented before the movement onset, but in 25% of the trials, it went to vertical orientation as soon as participants started to move. Tunik et al. [8] found that the unexpected perturbations in orientation had considerable effects on reach-to-grasp kinematics. The final metacarpophalangeal (MCP) joint angle and the peak MCP angle were significantly larger for the perturbed than the unperturbed trials. The time to peak MCP was also significantly delayed in the perturbed trials, and the adaptive responses occurred around 271 ms after the perturbation.

Individuals correct their movements not only in response to the perturbations in the movement effectors or target objects, but also to compensate for the perturbations in the anticipated action effects (action goals) [35,36]. In a grasp-to-place task [35], with a modified “S1–S2” paradigm, participants were asked to grip a horizontal cylinder (either overhand or underhand, free choice) and placed the left or right end of the cylinder into a target disk according to the visual stimulus (final action goal, S1). As soon as participants started their movements, a secondary stimulus (S2) was triggered, which indicated whether the intended action goal was perturbed (20% of the trials) or not (80% of the trials). Hughes et al. [35] found that when the intended action goals were perturbed, participants corrected their initial grasp postures during reaching to ensure a comfortable hand posture at the end of the object placing (end-state comfort), which resulted in a longer reach time and a shorter time to peak velocity during reaching. The corrections occurred either early (30% of the normalized reach time) or late (46% of the normalized reach time) in the reaching phase. Nevertheless, these studies [35,36] have been limited to the perturbations in the final action goals. Apart from the final action goal, the initial action goal (grip posture) is also crucial in planning and controlling manual actions.

Rosenbaum et al. [37] have proposed that manual actions are organized with a hierarchical motor plan in which both the initial goal (anticipated start posture) and the final goal (anticipated end posture) are located at the top level, and all the transitions between the initial and the final goals are located at the lower levels. Compared to the final goal, the initial goal acts as an immediate task demand in the execution of grasping movements, whereas the final goal acts as a remote (further) task demand. Therefore, if the unexpected perturbation in the initial goals occurs during reaching, it would have a stronger interference with motor execution than a perturbation in the final goals because the initial goal is the immediate (direct) action effect of reaching. However, it has also been argued that the final action goals are more important for planning and executing manual actions than the initial grip goals [38,39,40], and initial grip postures are selected on the basis of final task purposes in movement corrections [35,41]. Since there has been little discussion on the movement corrections with a perturbed initial goal, it is still difficult to conclude whether the perturbed initial goals have stronger interference with motor execution than the perturbed final goals or not.

In this study, we sought to investigate how unexpected perturbations in the initial or the final action goals influence the execution of grasp-to-rotate movements. To address this issue, we induced an unexpected perturbation in either the initial or the final action goals with the modified S1–S2 paradigm. Participants were cued by a visual stimulus (S1) with specified initial and final goals. When participants started their movements, a secondary stimulus (S2) was triggered, which indicated whether the anticipated initial and final goals were perturbed or not. When the goals were perturbed, participants were asked to correct their movements to comply with the corresponding perturbations. Electroencephalography (EEG) was recorded during the movement execution. The event-related potentials (ERP) and the subsequent source analyses were employed to distinguish the neural mechanisms underlying the movement corrections to adapt to the perturbations in the initial or the final goals. 

On the basis of previous studies [8,20,21,35,42], we hypothesized that the perturbations in the action goals interfere with the motor execution, which can be characterized as longer reach times, stronger anterior N2s (incompatibility detection), stronger anterior P3s (issued action inhibition), and larger late slow waves (new action implementation) in the goal-perturbed conditions versus the non-perturbed condition. Moreover, we also assumed that the perturbed initial goals have a stronger interference with the motor execution than the perturbed final goals, due to the fact that the initial goal (how to grip the handle) is the immediate demand for the grasping action and an unexpected change in the immediate demand may have a stronger interference than a change in the future demand (final goal). In this regard, we further hypothesized that reach times might be longer for the movement correction to adapt to perturbed initial goals than perturbed final goals. Neurophysiologically, we expected a stronger anterior P3, as well as more positive late slow waves, for the trials with perturbed initial goals than with perturbed final goals, which may reflect (prepared) action inhibitions (P3) and (new) action implementation (slow waves) in movement re-planning. 

## 2. Method

### 2.1. Participants

Twenty-four volunteers were initially recruited for this experiment. Four of them were discarded due to the EEG artifact, resulting in a final sample of 20 participants (mean age = 24.30 years; *SD* = 2.32; 11 females). All participants were right-handed (mean score = 90; SD = 14; Edinburgh Handedness Inventory [43]). All participants were with normal or corrected-to-normal vision and had no history of psychiatric or neurological impairments. All participants gave their written informed consent under the Declaration of Helsinki before they participated in the experiment, and the experimental protocol was approved by the ethics committee at Bielefeld University (EUB, No.2021-085).

### 2.2. Apparatus and Stimuli

**Figure 1:**
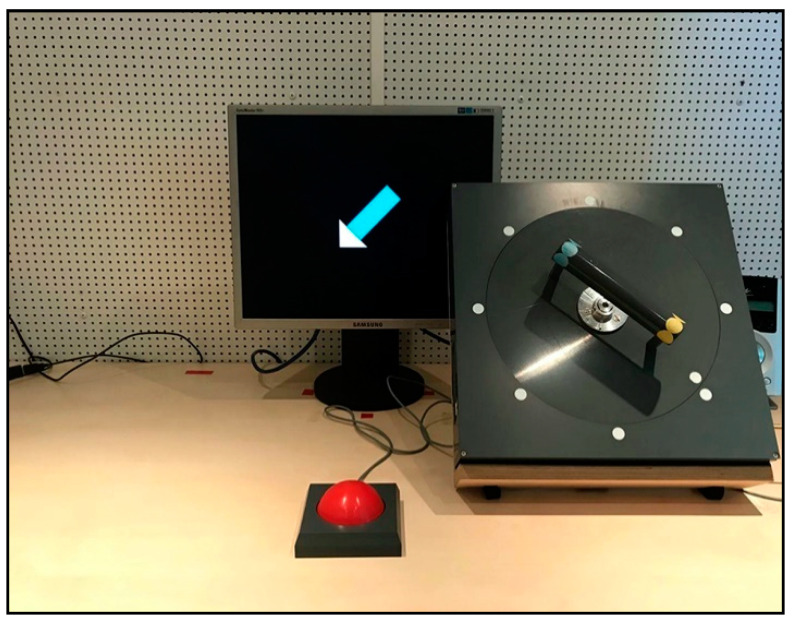
Front view of the experimental setup.

The setup of the grasp-to-rotate task is shown in Figure 1. The graspable part was a handle (cylinder, 16 cm in length, 3 cm in diameter) that had a yellow stripe at one end and a blue stripe at the other end. The handle was attached to a disk (28 cm in diameter), which could be rotated clockwise or counterclockwise. On the disk, a white pointing marker was placed next to the yellow end of the handle, and it was used to indicate the handle direction. Outside of the disk, eight target markers were fixed in the dial-display. During the experiment, the rotation apparatus was always settled where its center faced the shoulder of the participant’s grasping arm, and the distance was calibrated to each participant’s arm size for preventing expansive movements. A start button was placed in front of the participants, and the distance was also calibrated to each participant. The distance between the button and the rotation apparatus was constant for each participant (35 cm between the centers).

A 19-inch TFT monitor was placed behind the rotation apparatus, and it was about 75 cm away from the participants. Colored arrows were employed as the visual stimuli, and they were presented by Presentation (Neurobehavioral Systems, Berkeley, CA, USA). The color (yellow or blue) indicated that participants should grip the handle with their thumbs toward the corresponding stripe. The direction of the arrow (eight directions are identical with the eight target markers) indicated that participants should rotate the handle (pointing marker) to the pointed target marker. The direction was always perpendicular to the handle’s initial orientation, and participants were instructed to make 90-degree rotations. 

### 2.3. Experimental Paradigm

**Figure 2:**
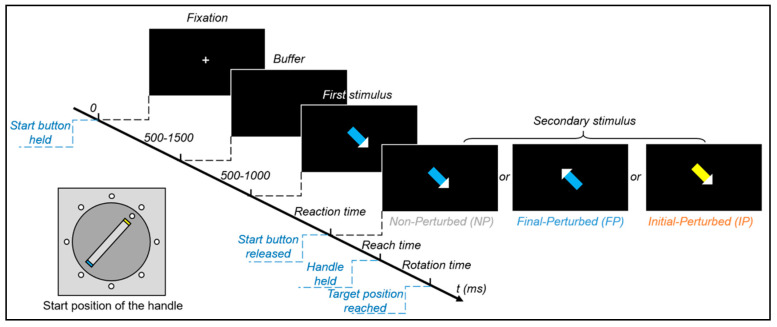
Time course of the task events. The time course of events during the experimental task with the example of a trial in which the handle starts at the northeast position. The trial started with a fixation cross with a variable time interval, followed by a buffer. After that, the first stimulus (S1) appeared, and it indicated the initial goal (blue) and final goal (the southeast direction) of the coming movement. A secondary stimulus (S2) was triggered as soon as participants started their movement. In the NP condition (**left**), the S2 was as same as the first. In the FP condition (**middle**), the final goal was perturbed (from the southeast to the northwest), but the initial goal stayed. In the IP condition (**right**), the initial goal was perturbed (from blue to yellow), but the final goal stayed. The S2 stayed on the screen until the handle reached the target position.

In this experiment, participants were instructed to grip the handle (initial goal) and rotate it to a target position (final goal). To introduce the movement correction, we used a modified S1–S2 paradigm. With the first stimulus (S1), the initial and the final goals were given by a colored arrow, and participants were instructed to respond to it. As soon as participants started their movements, another colored arrow was triggered as the secondary stimulus (S2), and it was either the same as or different from the first one. Participants were instructed to finish the movement with the new stimulus if it got perturbed. Thus, different S2s divided the experiment into three conditions (see Figure 2): (1) Final-Perturbed (FP)—the arrow direction changed to the opposite, but the color stayed; (2) Initial-Perturbed (IP)—the color changed to the opposite (yellow to blue or blue to yellow), but the direction stayed; (3) Non-Perturbed (NP)—neither color nor direction changed.

### 2.4. Procedure

After the EEG preparations, participants were guided to a shielded room and seated comfortably at the experimental desk. A written instruction was provided to the participants. The stimulus changes were not mentioned in the instruction for reducing the expectancy effect, and participants were only instructed to react to the stimulus, which was showing on the screen. All of the questions regarding the task were answered.

The experimental trial (see Figure 2) started with a voluntary button press. Then the handle automatically moved to the start position by the motor inside of the apparatus. The start positions were randomly assigned to each marker, and every marker had the same number of trials. After participants held the start button, a fixation cross was presented at the center of the screen with a variable duration from 500 to 1500 ms. After the fixation, a black screen was presented as a buffer for a variable duration from 500 to 1000 ms. Then the S1 was presented. Participants were asked to respond to it as soon as possible. Once the participants released the button, an S2 was presented. Participants had to correct their movements if the stimulus changed. The S2 disappeared when the handle reached the target position (precisely at the target marker). The next trial came after another button press. If the start button was released before the first stimulus, error feedback was presented for 1500 ms, and the next trial came after that. To minimize the ocular artifacts, participants were instructed to keep their gaze at the center of the screen during motor planning and execution. 

The experiment began with a practice section in which all 24 trials were non-perturbed trials. After the practice, participants started eight experimental blocks and each block contained 48 trials. Two-minute breaks were given between the blocks. To avoid the laterality of the brain activations due to hand use, we asked participants to perform the tasks with both hands (one hand for the first four blocks, and then change to the other hand). The starting hand (left/right) was counterbalanced. After the first four blocks, the rotation apparatus was moved to the other side and recalibrated. Half of the trials were gripped with the thumb toward the yellow stripe, and the remaining trials were gripped with the thumb toward the blue stripe. Half of the trials were rotated clockwise, and the remaining trials were rotated counterclockwise. The grips and rotation directions were randomly assigned to the trials. Additionally, to minimize the participant’s expectancy, we set the ratio of “FP/IP/NP” to 1/1/6, that is, 48 FP trials, 48 IP trials, and 288 NP in total. After the experiment, subjective difficulty ratings (from 1 to 6, from easy to difficult) for different perturbed conditions were queried. It took around 2 h to finish the experiment. 

### 2.5. Behavioral and Electrophysiological Recordings

The participants’ performance was recorded by a video camera. The time points of releasing the start button, gripping the handle, and reaching the target position were detected by the micro-switches in the apparatus. The reaction time (from the first stimulus onset to movement onset), reach time (from movement onset to gripping the handle), and rotation time (from gripping the handle to reaching the target position) were calculated with these time points. 

The electroencephalography (EEG) signals were collected by an ANT amplifier and the acquisition software ASA (ANT Neuro, Hengelo, The Netherlands) at a sampling rate of 512 Hz. Recordings were made from 64 Ag/AgCl electrodes, which were positioned in accordance with the international 10–10 system. Electrooculography (EOG) was also recorded by two bipolar electrodes placed above and below the right eye and lateral to both eyes. The impedance of all electrodes was less than five kΩ, and the electrode AFz was selected as the recording ground. All signals were band-pass filtered (DC-138 Hz) and average-referenced during the recording.

### 2.6. Data Analysis

#### 2.6.1. Behavioral Data 

Based on the performance videos, trials with wrong grips, wrong rotation directions, or changing grip during the rotation were excluded from the behavioral and neurophysiological analyses. Trials with extreme (outside of mean ± three standard errors) reaction time, reach time, or rotation time were also excluded. On average, participants executed the task correctly in 85% of the FP trials, 84% of the IP trials, and 93% of the NP trials. Since we did not find any significant main or interaction effects involving “hand used” in behavioral timings, we pooled the left- and right-hand trials together in the analysis. The average numbers (and the standard deviations) of the remaining trials in different conditions are shown in Supplementary Table S1. Repeated-measures ANOVAs were performed separately on participants’ averaged reaction times, reach times, and rotation times to determine the within-subject effect for *perturbation* (FP/IP/NP). 

#### 2.6.2. ERPs 

EEG signals were offline analyzed with the toolbox EEG lab [44] and ERP lab [45]. All signals were band-pass filtered (0.1–30 Hz) and re-referenced with the linked mastoid electrodes. Two analysis epochs were extracted from the continuous signals. Epoch time-locked to S2 (as well as movement onset) included the time interval from −1400 to 1000 ms. Epoch time-locked to grasping included the time interval from −2900 to 300 ms. Baseline correction was performed with the first 300 ms of the epochs. Gratton regression [46,47] was employed to correct the ocular artifacts. Any trials containing peak-to-peak amplitudes above 100 µV within a moving window (200 ms window; 50 ms step) were automatically removed. The remaining trials were visually double-checked for artifacts that would not have been detected by the moving window algorithm. On average, there were 34 FP trials, 35 IP trials, and 232 NP trials left for averaging the ERPs in the epoch time-locked to S2, and there were 33 FP trials, 34 IP trials, and 227 NP trials left for averaging the ERPs in the epoch time-locked to grasping (see Supplementary Table S1 for more details).

In the epoch time-locked to S2, an obvious P3 (300–600 ms) and obvious late positive slow-wave potentials (600–1000 ms) were found for the goal-perturbed conditions (FP, IP), as compared to the NP condition. For the P3 component, the amplitude was quantified as the mean amplitude from 390 to 440 ms (the average P3 peak latency was 415 ms). For the slow-wave potentials, mean amplitudes were measured and compared in 100 ms step windows. Both the P3 and slow-wave potentials were accessed among nine regions of interest (ROI) to assess the scalp distribution. The ROIs were anterior-left (AL): AF7, F7, F5, F3; anterior-middle (AM): F1, Fz, F2; anterior-right (AR): AF8, F8, F6, F4; central-left (CL): C3, C5, CP3, CP5; central-middle (CM): FCz, Cz, CPz; central-right (CR): C4, C6, CP4, CP6; posterior-left (PL): PO7, PO5, PO3, O1; posterior-middle (PM): Pz, POz, Oz; posterior-right (PR): PO8, PO6, PO4, O2. Repeated-measures ANOVAs with the factor *perturbation* (FP/IP/NP), *left–right* (left/middle/right), and *front–back* (anterior/central/posterior) were performed on the mean amplitudes of the electrodes (in corresponding ROIs).

In the epoch time-locked to grasping, we only focused on the slow-wave potentials before grasping. According to the previous findings [39,48,49], the analysis time window was set as −500–0 ms. Similar to the previous epoch, the mean amplitudes of the slow-wave potentials time-locked to grasping were also compared in 100 ms step windows. Mean amplitudes of the above-mentioned nine ROIs were compared by repeated-measures ANOVAs with the factor *perturbation* (FP/IP/NP), *left–right* (left/middle/right), and *front–back* (anterior/central/posterior) to determine the perturbation effect and its scalp distribution. 

All the above-mentioned ANOVAs were conducted in R [50]. Greenhouse-Geisser correction was applied whenever the sphericity assumption was violated. The original degrees of freedom and the corrected *p*-values were reported. Generalized eta-squared ($\eta_{G}^{2}$) was used for evaluating the effect size. Post hoc multiple comparisons among means were made with Bonferroni *t*-tests.

#### 2.6.3. Source Analysis 

In a subsequent analysis, the three-dimensional cortical distributions of the averaged ERPs (in different conditions) were analyzed with the standardized low-resolution brain electromagnetic tomography analysis software (sLORETA) [51]. The sLORETA partitions the intracerebral volume in 6239 grey matter voxels with a spatial resolution of 5 mm, and the standardized scalp current density at each voxel is then calculated in a realistic head model [52] with the probabilistic MNI152 template [53]. 

In order to identify possible differences in the brain electrical activity between the goal-perturbed conditions (FP vs. IP), statistical non-parametric mapping (SnPM) [54] was employed for computing the averaged intracerebral current density distribution at the time intervals showing significant differences based on a non-parametric *log-F*-ratio statistic on the three-dimensional sLORETA images (number of randomizations = 5000). The SnPM corrected for multiple comparisons [54]. Voxels with significant differences (*p* < 0.05) between the perturbed conditions were located in specific brain regions with Brodmann areas (BA) and the MNI coordinates.

## 3. Results

### 3.1. Subjective Difficulty Ratings 

Participants rated the subjective difficulty of FP trials as 3.20 (*SD* = 1.36) on a scale from 1 (easy) to 6 (difficult). For the IP trials, the average difficulty was rated as 3.35 (*SD* = 1.18). The paired *t*-test yielded that the subjective difficulty was not significantly different between the goal-perturbed conditions, *t*(19) = 0.65; *p* > 0.05. 

### 3.2. Timing

**Figure 3:**
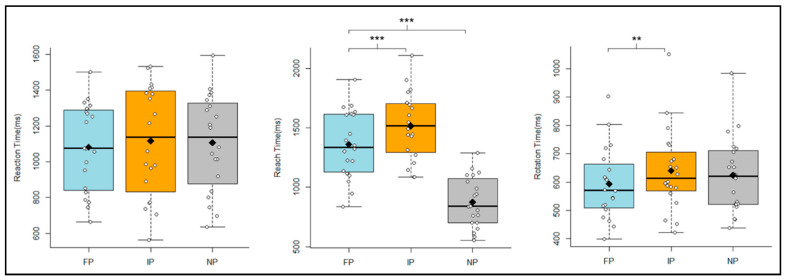
Timing of behavior. Box plots representing reaction times (**left**), reach times (**middle**), and rotation times (**right**) of the 20 participants. Light blue boxes show the data for the Final-Perturbed (FP) condition. Orange boxes show the data for the Initial-Perturbed (IP) condition. Grey boxes show the data for the Non-Perturbed (NP) condition. The black rhombuses represent the means. The “***” stands for *p* <0.001, and the “**” stands for *p* < 0.01.

The averaged reaction, reach, and rotation times for different conditions were shown in Figure 3. For the reaction time, we did not find a significant difference among the different conditions (FP/IP/NP), *F*(2,38) = 2.69; *p* > 0.05. 

For the reach time, we found a significant main effect for *perturbation*, *F*(2,38) = 132.52; *p* < 0.001; $\;\eta_{G}^{2}$ = 0.533. Post hoc analyses revealed that the reach time was longer in the IP condition (1515 ms, *SD* = 281) than the FP condition (1359 ms, *SD* = 285), *t*(19) = 7.59; *p* < 0.001, and a longer reach time was also found for the FP condition than the NP condition (875 ms, *SD* = 213), *t*(19) = 10.58; *p* < 0.001.

For the rotation time, we also found a significant main effect for *perturbation*, *F*(2,38) = 7.13; *p* < 0.01; $\;\eta_{G}^{2}$ = 0.021. Post hoc analyses yielded that rotation times were significantly longer for the IP condition (641 ms, *SD* = 145) than for the FP condition (593 ms, *SD* = 126), *t*(19) = −3.73; *p* < 0.01. However, the difference between the NP condition (624 ms, *SD* = 136) and the FP condition was not significant, *t*(19) = 2.26; *p* > 0.05, and neither was the difference between the NP condition and the IP condition, *t*(19) = −1.37; *p* > 0.05.

### 3.3. ERP Results

#### 3.3.1. Epoch Time-Locked to the Secondary Stimulus

**Figure 4:**
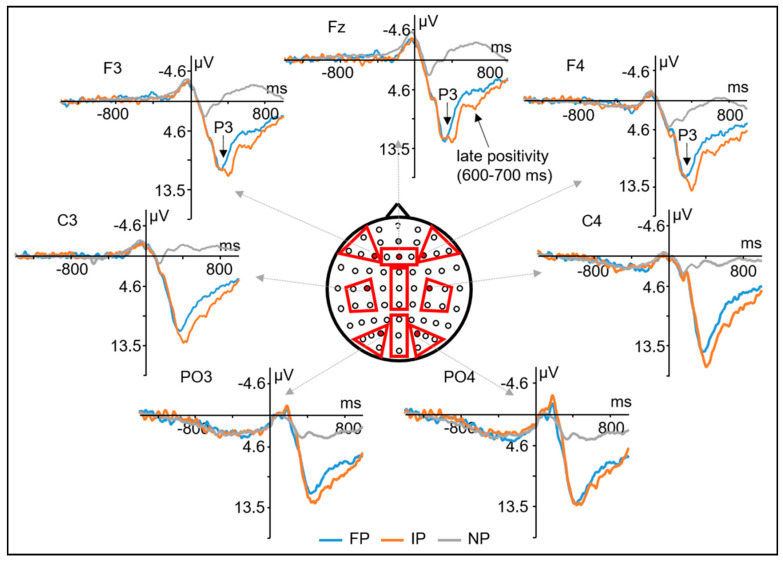
Grand-averaged ERP waveforms time-locked to S2. Grand-averaged ERP waveforms (N = 20) recorded time-locked to S2 onset, for the Final-Perturbed condition (blue), Initial-Perturbed condition (orange), and Non-Perturbed condition (grey) are showed at one anterior-left electrode (F3), one anterior-middle electrode (Fz), one anterior-right electrode (F4), one central-left electrode (C3), one central-right electrode (C4), one posterior-left electrode (PO3), and one posterior-right electrode (PO4). The nine regions of interest (ROIs) for ERP analysis are marked with red color in the middle head plot.

P3 (300–600 ms) With the factor *perturbation* (FP/IP/NP), *left–right* (left/middle/right), and *front–back* (anterior/central/posterior), the ANOVA of P3 amplitude yielded significant interaction effects for perturbation*front–back, *F*(4,76) = 9.83; *p* < 0.001; $\eta_{G}^{2}$ = 0.015, perturbation*left–right, *F*(4,76) = 25.24; *p* < 0.001; $\eta_{G}^{2}$ = 0.010, and left–right*front–back, *F*(4,76) = 9.98; *p* < 0.001; $\eta_{G}^{2}$ = 0.007. We also found significant main effects for *perturbation*, *F*(2,38) = 39.27; *p* < 0.001; $\eta_{G}^{2}$ = 0.268, and *left–right*, *F*(2,38) = 20.16; *p* < 0.001; $\eta_{G}^{2}$ = 0.017. Further analyses revealed that in the anterior ROIs, P3 mean amplitude was larger in the IP condition (11.53 μV, *SD* = 8.16) than the FP condition (8.10 μV, *SD* = 6.29), *t*(19) = 3.22; *p* < 0.01, and the P3 in FP condition was also larger than the NP condition (0.80 μV, *SD* = 7.34), *t*(19) = 6.87; *p* < 0.001. However, in the central and posterior ROIs, the amplitude differences between the IP and FP conditions were not significant, all *t*s < 1.62; all *p*s > 0.34, but the P3 amplitudes were larger in the goal-perturbed conditions (FP, IP) than the non-perturbed condition (NP), *t*s > 4.72; all *p*s < 0.001 (see Supplementary Table S2 for more details). Moreover, the P3 amplitudes were also larger in the goal-perturbed conditions (FP, IP) than the non-perturbed condition (NP), *t*s > 5.94; all *p*s < 0.001, over the left, middle, and right ROIs. Nevertheless, the amplitude differences between the IP and FP conditions were not significant, all *t*s < 1.46; all *p*s > 0.46. No other significant effects between the FP and IP conditions were found in the further analyses. The ERP waveforms can be seen in Figure 4.

**Figure 5:**
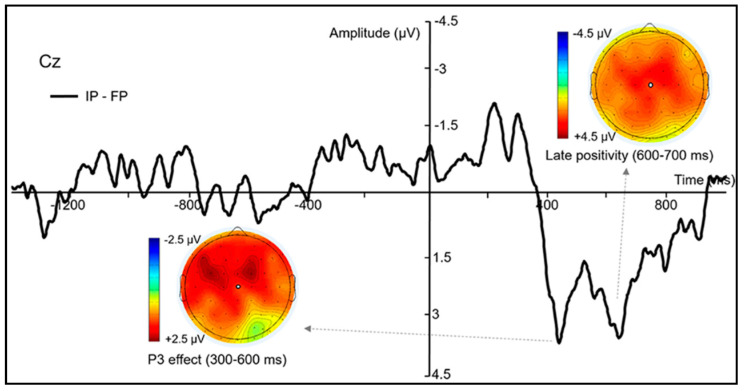
The ERP difference waves time-locked to S2. ERP difference waves (“Initial-Perturbed” –“Final-Perturbed”) time-locked to the onset on S2 at the central-middle electrode (Cz). Topographical maps of the difference waves in the P3 (300–600 ms) and late positivity (600–700 ms) time intervals are also showed.

Slow-wave potentials (600–1000 ms) For the ERP slow-wave potentials from 600 to 700 ms, the ANOVA revealed a significant interaction effect for perturbation*left–right, *F*(4,76) = 8.71; *p* < 0.001; $\eta_{G}^{2}\;$= 0.002, and a significant interaction effect for front–back*left–right, *F*(4,76) = 6.16; *p* < 0.001; $\eta_{G}^{2}\;$= 0.004. The main effects for *perturbation*, *F*(2,38) = 36.12; *p* < 0.001; $\eta_{G}^{2}\;$= 0.110, *front–back*, *F*(2,38) = 11.70; *p* < 0.001; $\eta_{G}^{2}\;$= 0.018, and *left–right*, *F*(2,38) = 17.87; *p* < 0.001; $\eta_{G}^{2}\;$= 0.013, were also significant. To explain the significant interaction for perturbation*left–right, we conducted further analyses, and the results revealed that the amplitudes of the slow-wave potentials were larger for the goal-perturbed conditions (FP and IP) than the NP condition in the left, middle, and right ROIs (all *t*s > 4.95; all *p*s < 0.001) (see Supplementary Table S3 for more details). However, the amplitude difference between the IP and the FP conditions was only significant in the middle ROIs. In the middle ROIs, the mean amplitude of the slow-wave potentials was larger in the IP condition (7.85 μV, *SD* = 10.20) than the FP condition (5.27 μV, *SD* = 8.38), *t*(19) = 2.52; *p* < 0.05. The difference waves between FP and IP conditions, as well as the topographic maps of the difference waves, are shown in Figure 5.

For the slow-wave potentials in the time windows from 700 to 800 ms, from 800 to 900 ms, and from 900 to 1000 ms, all the ANOVAs revealed significant main effects for *perturbation*, all *F*s > 6.55; all *p*s < 0.01. Nevertheless, the interaction effects for perturbation*front–back, perturbation*left–right, and perturbation*front–back*left–right were all not significant in these time windows (see Supplementary Table S4 for more details). Post hoc analyses revealed that the mean amplitudes of the slow-wave potentials were more positive for the goal-perturbed conditions (FP, IP) than the non-perturbed condition in all the time windows, all *t*s > 2.89; all *p*s < 0.05, whereas the mean amplitudes were not different significantly between the FP and IP conditions, all *t*s < −1.43; all *p*s > 0.48 (see Supplementary Table S5 for more details).

#### 3.3.2. Epoch Time-Locked to Grasping

For the epoch time-locked to grasping, we compared the slow-wave potentials from −500 to 0 ms in 100 ms step windows. For the time window from −500 to −400 ms, the ANOVA yielded a significant main effect for *perturbation*, *F*(2,38) = 3.79; *p* < 0.05; $\eta_{G}^{2}\;$ = 0.014, a significant main effect for *front–back*, *F*(2,38) = 3.79; *p* < 0.05; $\eta_{G}^{2}\;$ = 0.014, a significant main effect for *left–right*, *F*(2,38) = 29.89; *p* < 0.001; $\eta_{G}^{2}\;$ = 0.031, and a significant interaction effect for front–back* left–right, F(4,76) = 4.23; *p* < 0.05; $\eta_{G}^{2}\;$ = 0.005. Post hoc analyses found that the slow-wave potentials were more positive in the FP (4.86 μV) than the NP (2.70 μV) condition, *t*(19) = 2.73; *p* < 0.05. However, the amplitude difference was not significant neither between the FP and IP conditions, *t*(19) = 1.05; *p* > 0.05, nor between the IP and NP conditions, *t*(19) = 1.68; *p* > 0.05.

**Figure 6:**
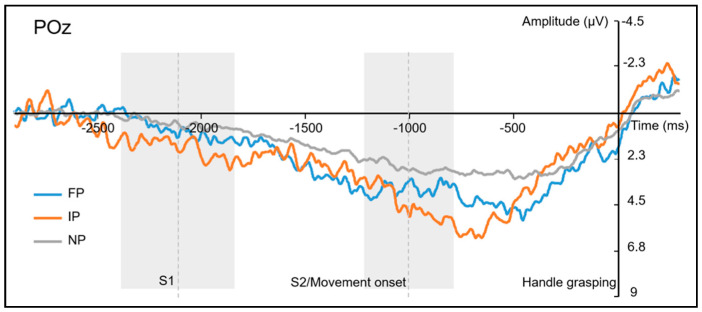
Grand-averaged ERP waveforms time-locked to grasping at electrode POz. Grand-averaged ERP waveforms (N = 20) recorded at electrode POz, time-locked to grasping, for the Final-Perturbed condition (blue), Initial-Perturbed condition (orange), and Non-Perturbed condition (grey). Average time points (dash lines) for the first stimulus presentation (S1), the secondary stimulus (S2) presentation/movement onset are marked (shaded areas beside the dash lines indicate the standard deviations).

For the slow-wave potentials in the time windows from −400 to −300 ms, from −300 to −200 ms, from −200 to −100 ms, and from −100 to 0 ms, the ANOVAs found no significant main effects for, all *F*s < 2.09; all *ps* > 0.14, no significant interactions for perturbation*front–back, all *F*s < 1.24; all *ps* > 0.30, no significant interactions for perturbation*left–right, all *F*s < 1.35; all *ps* > 0.27, and no significant interactions for perturbation*front–back*left–right, all *F*s < 1.00; all *ps* > 0.37 (see Supplementary Table S6 for more details). The ERPs time-locked to grasping are shown in Figure 6.

### 3.4. Source Analysis

**Figure 7:**
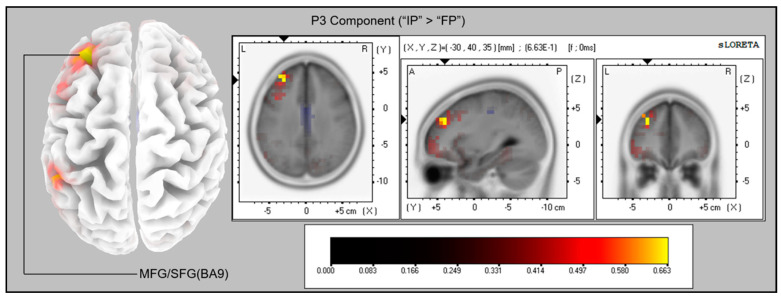
Results of the sLORETA source analysis (contrast: “Initial-Perturbed” > “Final-Perturbed”) in the time window of the P3 component (300–600 ms). The images have been obtained after statistical non-parametric mapping (SnPM), and they represent the voxels in which the “Initial-Perturbed” > “Final-Perturbed” contrast was significant (*p* < 0.05; *log-F* threshold = 0.650) in the time window of the P3 component. The significantly activated voxels are indicated by yellowish colors.

For the averaged time window between 390 and 440 ms time-locked to S2 (corresponding to the P3 component), a significantly higher cortical activation for the IP in contrast to the FP conditions was found in the following cortical areas: the left middle frontal gyrus (MFG; BA9; *x* = −30, *y* = 40, *z* = 35, and *x* = −30, *y* = 40, *z* = 30), and the left superior frontal gyrus (SFG; BA9; *x* = −35, *y* = 45, *z* = 30). The maximum difference was located at the left MFG, *log-F* = 0.663, *p* < 0.05 (*log-F* threshold = 0.650; see Figure 7).

For the averaged time window between 600 and 700 ms time-locked to S2 (corresponding to the late positivity from 600 to 700 ms), we found the cortical activation difference between the goal-perturbed conditions were mainly located at the cingulate gyrus (BA24; x = −10, y = 0, z = 45), and the medial frontal gyrus (MFG; BA6; x = −5, y = −25, z = 70). However, the activation difference in neither of the areas reached the significance level: all *log-F*s < 0.470, all *p*s > 0.05 (*log-F* threshold = 0.488). 

## 4. Discussion

In this experiment, we examined how unexpected perturbations in initial or in final action goals interfere with the execution of grasp-to-rotate movements and the neural mechanisms underlying the adjustments in response to the goal perturbations. The results revealed that compared to a perturbed final goal, a perturbed initial goal significantly slowed down the movement execution. Moreover, a larger frontal P3 and larger central-distributed late positivity (600–700 ms) time-locked to the perturbations were found for the initial than for the final goals. Further source analyses suggested increased cortical activations in the left middle frontal gyrus (MFG, BA9) and left superior frontal gyrus (SFG, BA9) were found for the perturbed initial goals than the perturbed final goals in the P3 time window. Taking together, these findings suggest the influence of perturbed initial and final action goals in the execution of grasp-to-rotate movements differs, and the unexpected perturbations in initial goals seem to have stronger interference with motor execution than final action goals.

Participants rated the subjective difficulty in the FP condition with 3.20 and in the IP condition with 3.35, on a scale from 1 (easy) to 6 (difficult). Even though the participants rated the IP condition slightly harder than the FP condition, the difference was not significant. Participants perceived similar subjective difficulties for the goal perturbations. It seems to be in line with our accuracy results that participants executed the task correctly in 85% of trials in the FP condition and 84% of trials in the IP condition. These indicate that task difficulty did not differ between the perturbation conditions and, hence, task difficulty is unlikely to be related to any effects found between the FP and IP conditions.

As expected, reaction times (from fist stimulus onset to movement onset) were not different among the conditions (FP/IP/NP). Reaction times usually reflect the motor planning processes before movement onset [55,56]. In our experiment, reaction times reflect the movement preparations for the first stimuli. Since the first stimuli were not perturbed, therefore, reaction times should be similar among the conditions.

Consistent with the previous findings [35], as well as our hypothesis, reach times (from movement onset to holding the handle) in the goal-perturbed conditions (FP and IP) were significantly slower than the non-perturbed condition (NP). The prolonged reach time reflected the movement corrections compensating for the perturbations in action goals. More importantly, we found reach times were significantly slower in the IP than in the FP condition. It seems to indicate that the perturbations in the initial action goals have stronger interference with the correction of manual actions as compared to the perturbations in the final action goals, and the stronger interference slows down the reaching movements. 

For the rotation time (from holding the handle to reaching the target marker), the difference between the FP and the NP conditions was not significant, nor was the difference between the IP and the NP conditions. These findings are consistent with previous studies [35,36], which revealed that object manipulation times are not influenced by the perturbation in the anticipated action goal. 

Interestingly, the rotation times were significantly different between the goal-perturbed conditions. Participants moved the handle much slower in the IP than in the FP condition. A possible explanation can be that the perturbations in the initial goals may increase the participant’s awareness of potential grip errors, even if the grip has already been performed. In the IP condition, the initial goals were changed, and the perturbation made participants drive more attention toward their grip postures. So participants might always have “double-checked” their movements (grip postures) to avoid a potential error, even after the handle had been gripped. 

With a bar transport task, Westerholz et al. [39] found that the transport times (durations between grasping and bar-on-target) were slower when the initial goals were emphasized (compared to when the final goals were emphasized), even though the same movements were performed in both goal-emphasized conditions. The “initial-emphasized” seems to be similar to the current IP condition in which participants may focus on the perturbed initial goals, and the “final-emphasized” seems to be similar to the current FP condition in which participants may focus on the perturbed final goals. Moreover, in another study with a similar grasp-to-rotate task (Yu et al., under review), we also found that the rotation times were slower when the initial goals were perturbed unexpectedly (during movement preparation), as compared to when the final goals were perturbed unexpectedly. Therefore, the prolonged rotation times might be attributed to participants’ increased awareness of potential grip errors.

As for the ERPs, we did not find the expected N2 effect between the goal-perturbed and the non-perturbed conditions. From the grand-averaged ERPs shown in Figure 4, we can hardly tell an obvious N2 component around 200 ms over the anterior area (except for a slight negative-going oscillation). The reduction of anterior N2 can be attributed to the overlapping of N2 and P3 components. Because the probabilities of the different S2s were unequal, large anterior P3s were evoked in the goal-perturbed trials. The large P3s may overlap and reduce the observed N2 amplitude. Moreover, Kraemer et al. [57] also reported that changing a response may not elicit an anterior N2 component as compared to stopping a prepared response.

Consistent with our hypothesis, we found that the mean amplitude of the P3 component (time-locked to S2) was significantly larger for the goal-perturbed conditions (FP and IP) than the non-perturbed condition (NP). This is consistent with previous findings that movement corrections elicited stronger P3s than the movement execution without correction [20,21,58]. The increased P3 activities in the goal-perturbed conditions reflect the inhibitory processes to stop inappropriate actions compensating for the perturbations in the action goals. Additionally, the enlarged frontal P3s in the goal-perturbed conditions can also be attributed to the stimulus novelty. Compared to the NP condition, the perturbed stimuli in the goal-perturbed conditions are infrequent. The infrequent stimuli may also elicit larger frontal P3 compared to the frequent stimuli [59,60]. 

More importantly, the mean amplitude of P3 was larger in the IP than in the FP condition over the anterior ROIs. From the topographic map shown in Figure 5, it can be seen that the amplitude difference maximizes over the left and the middle frontal areas. It is also further confirmed by the source analyses results that activations of the left middle frontal gyrus (MFG, BA9) and left superior frontal gyrus (SFG, BA9) were significantly higher in the IP than in the FP condition. It has been reported that in the go/no-go task [61,62,63,64,65,66,67], the stop-signal task [68,69,70,71,72,73,74], and the movement re-planning tasks [20,21,58], larger P3s were evoked over the frontal and central areas when the responses had been inhibited or corrected in comparison to those that were normally executed. Previous studies also suggest that MFG seems to be one of the neural generators for the frontal P3 component evoked by response inhibitions [75,76]. Previous neuroimaging studies have claimed that the left MFG is involved in inhibitory processing, and stronger MFG activations were found when a prepared response was stopped [77,78,79,80,81,82]. Moreover, the left dorsolateral prefrontal cortex (DLPFC), which lies in the left MFG, has been associated with inhibiting the stereotyped responses [83] or processing incongruous object–action combinations [84]. Therefore, the enlarged P3 amplitude and the stronger activations of the left MFG in the IP condition compared to the FP condition may indicate that perturbations in initial goals induce a stronger inhibition process during the movement correction in which participants are trying to stop the inappropriate actions to prevent potential errors.

It is interesting that we only found different cortical activations in the left hemisphere between the FP and IP conditions, even though both left- and right-hand movements were performed and averaged in our study. It seems to be consistent with the idea that the left hemisphere is specialized for motor planning [85,86,87,88,89]. A recent fMRI study [90] also suggested that the left hemisphere (left-SMA) plays a critical role in interhemispheric inhibition and motor planning. However, several studies [10,15,17] have reported that several right-hemisphere regions, such as the right-SMA, are associated with motor inhibitions and movement selections during motor re-programming (re-planning). It is still hard to conclude whether brain lateralization exists in motor re-planning or not. For future research, it might be of interest to focus on brain lateralization in motor re-planning. 

For the late slow-wave potentials (600–1000 ms) time-locked to S2, we found that the mean amplitudes were significantly larger in the goal-perturbed conditions (FP, IP) than the non-perturbed condition (NP), which is also consistent with our hypothesis. The enlarged slow-wave potentials may reflect the increased cognitive efforts involved in the action implementations or action reorganizations during the movement corrections. Between the different goal-perturbed conditions, the mean amplitudes were significantly larger for the IP than for the FP condition only in the time window from 600 to 700 ms (time-locked to S2), and the difference was only found in the middle ROIs. From the topographic map in Figure 5, we can see the difference waves maximize over the frontocentral areas. Further source analyses also yielded higher but not significant activations around the cingulate gyrus for the IP than for the FP condition. Moreover, from the averaged ERP waveforms in Figure 4, an obvious positive component can be found in the IP condition, which peaks around 600 ms after the S2 onset. This late positivity is reminiscent of the P600 effect obtained in language studies, which reflects the processing of structured representations at the syntactic level [91,92,93,94,95,96,97]. The P600 is elicited when there is a syntactic violation in a sentence, and it is characterized for the reanalysis or repair of the sentence structure. Some studies have also reported that the P600 was evoked by the violation of action [98,99] or music structures [100,101]. Therefore, the enlarged P600-like late positivity (slow-wave potential) elicited by the perturbed initial goals may reflect the increased demands on action restructuring or reorganization in the correction of ongoing manual actions. Compared to perturbed final goals, movement corrections to adapt to perturbed initial goals seem to require more effort in the implementation of new actions.

For the slow-wave potentials before grasping (from −500 to 0 ms time-locked to grasping), we only found a significant amplitude difference between the FP and the NP conditions in the time window from −500 to −400 ms, and the slow-wave potentials were more positive in the FP than the NP condition. However, the difference between the FP and the IP was not significant, neither was the difference between the IP and the NP. Considering the temporal overlapping of the epochs (time-locked to S2 and time-locked to grasping), the enlarged slow-wave potentials for the FP condition in the time window (−500–400 ms) might be attributed to the movement correction processes (the late positivity potentials in the epoch time-locked to S2). It is worth noting that the difference in slow waves might also be attributed to eye movements. Even though we instructed participants to fix their gazes during movement and corrected the ocular artifacts by Gratton regression [46,47], the (potential) residual effects might still remain (especially) in a large analysis epoch, which could influence (partially) the slow-wave amplitude. Therefore, the slow-wave effect between FP and NP conditions might also be caused by the residual effects of eye movement. It is still an open question, which deserves further research.

In the time windows from −400 to 0 ms time-locked to grasping, no significant difference was found for the slow-wave potentials among the different conditions (FP/IP/NP). It seems to suggest that participants adjust their movements as soon as they perceive the perturbations, and the movement corrections may have been finished at least 400 ms before grasping. It is in line with the movement kinematics data in the previous study [35] that movement correction to adapt to a perturbed (final) action goal occurred in the first half of the reach time. More importantly, the similar slow-wave potentials before grasping between the FP and the IP also indicate that the (neuro-) cognitive processes before grasping are very similar for both goal-perturbed conditions, which may exclude the possibility that participants corrected their movements only after they gripped the handle in the FP condition.

Limitations of the present study should be taken into consideration. Even though none of the participants reported (in the post questionnaire) a strategy that they planned all of the four possible grasp-to-rotate movements before they release the start button, it still could be a limitation for our experimental design (the corrections of the upcoming movements are predictable) and it may affect our results (such as the reaction times). For future research, it would be interesting if unpredictable changes can be involved. To control the length of the experiment, we did not include a third “perturbed” condition in which both initial and final action goals are perturbed. Future research may consider implementing this in order to provide a more comprehensive picture of goal perturbations in manual actions. 

## 5. Conclusions

Taking the behavioral and neurophysiological results together, we found that the re-planning times, as well as the cortical activities, differed between the corrections of manual actions with perturbed initial goals and perturbed final goals. The perturbed initial goals have a stronger interference with the execution of the grasp-to-rotate movement than the perturbed final goals, and the interference seems to arise from both motor inhibition (stopping inappropriate actions) and motor implementation (generating new actions). To our knowledge, this is one of the first studies to distinguish the online corrections of manual actions with perturbed initial and final action goals, as well as the first study to differentiate cerebral activity underlying overt goal-related manual actions executed with an unexpectedly perturbed initial and final action goal. Our findings emphasized the importance of the initial action goals (grip postures) in the execution and online correction of manual actions; the unexpected changes in the required grip postures seem to demand more efforts in both action inhibitions and implementations (compared to changes in task purposes).

## Data Availability

Relevant data are within the manuscript and its Supplementary Materials. Additional relevant data (i.e., EEG raw data) are available at https://doi.org/10.4119/unibi/2952950.

## Supplementary Materials

The following are available online at https://www.mdpi.com/article/10.3390/brainsci11050641/s1, Table S1: The average number (standard deviation) of trials for analysis in different experimental conditions; Table S2: Simple effect of perturbation for P3 amplitude in different front-back and left-right areas; Table S3: Simple effect of perturbation for the mean amplitude of the slow waves during 600–700 ms in different left–right areas; Table S4: Summary of the statistical results for the slow waves from 700 to 1000 ms (time-locked to S2); Table S5: Summary of the post hoc results for the factor perturbation from 700 to 1000 ms (time-locked to S2); Table S6: Summary of the statistical results for the ERP slow waves from −400 to 0 ms (time-locked to grasping)
