# Supplementary material for: Online Movement Correction in Response to the Unexpectedly Perturbed Initial or Final Action Goals: An ERP and sLORETA Study
Source: Brain Sci. 2021 May 15;11(5):641. doi: 10.3390/brainsci11050641 (PMC8156469; doi:10.3390/brainsci11050641)
Supplement: Supplementary file 1 [file brainsci-11-00641-s001.zip › brainsci-1176184-supplementary/Table S1.pdf]

Supplementary Table S1 The average number (standard deviation) of trials for analysis in different experimental conditions

	FP	***IP***	***NP***
**Behavioral Timing**	40(3.85)	41(3.05)	268(7.48)
**Time-locked to the S2**	34(3.62)	35(3.10)	231(24.50)
**Time-locked to grasping**	33(5.70)	34(5.27)	227(22.02)
