# Supplementary material for: Online Movement Correction in Response to the Unexpectedly Perturbed Initial or Final Action Goals: An ERP and sLORETA Study
Source: Brain Sci. 2021 May 15;11(5):641. doi: 10.3390/brainsci11050641 (PMC8156469; doi:10.3390/brainsci11050641)
Supplement: Supplementary file 1 [file brainsci-11-00641-s001.zip › brainsci-1176184-supplementary/Table S2.pdf]

Supplementary Table S2 Simple effect of *perturbation* for P3 amplitude (time-locked to S2) in different front–back and left–right areas

Area		RM ANOVA		Post Hoc	
		F	***η_G^2***	**Contrast**	***t***
				FP−IP	−3.42**
	**Anterior**	53.096***	0.283	FP−NP	6.87***
				IP−NP	10.09***
				FP−IP	−1.62
**Front–back**	**Central**	46.38***	0.345	FP−NP	7.41***
				IP−NP	9.03***
				FP−IP	−0.48
	**Posterior**	16.53***	0.19	FP−NP	4.72***
				IP−NP	5.20***
				FP−IP	−1.61
	**Left**	32.28***	0.251	FP−NP	6.01***
				IP−NP	7.62***
				FP−IP	−1.86
**Left–right**	**Middle**	50.52***	0.357	FP−NP	7.63***
				IP−NP	9.49***
				FP−IP	−1.46
	**Right**	30.72***	0.249	FP−NP	5.94***
				IP−NP	7.40***

Note: * $p < 0.05$; ** $p < 0.01$; *** $p < 0.001$
