# Supplementary material for: Online Movement Correction in Response to the Unexpectedly Perturbed Initial or Final Action Goals: An ERP and sLORETA Study
Source: Brain Sci. 2021 May 15;11(5):641. doi: 10.3390/brainsci11050641 (PMC8156469; doi:10.3390/brainsci11050641)
Supplement: Supplementary file 1 [file brainsci-11-00641-s001.zip › brainsci-1176184-supplementary/Table S3.pdf]

Supplementary Table S3 Simple effect of *perturbation* for the mean amplitude of the slow waves during 600–700 ms (time-locked to S2) in different left−right areas

Area		RM ANOVA		Post Hoc	
		F	***η_G^2***	**Contrast**	***t***
**Left−right**	**Left**	36.97***	0.125	FP−IP	−2.19
				FP−NP	6.10***
				IP−NP	8.30***
	**Middle**	41.28***	0.141	FP−IP	−2.52*
				FP−NP	6.30***
				IP−NP	8.82***
	**Right**	25.76***	0.085	FP−IP	−2.03
				FP−NP	4.95***
				IP−NP	6.98***

Note: * $p < 0.05$; ** $p < 0.01$; *** $p < 0.001$
