# Supplementary material for: Online Movement Correction in Response to the Unexpectedly Perturbed Initial or Final Action Goals: An ERP and sLORETA Study
Source: Brain Sci. 2021 May 15;11(5):641. doi: 10.3390/brainsci11050641 (PMC8156469; doi:10.3390/brainsci11050641)
Supplement: Supplementary file 1 [file brainsci-11-00641-s001.zip › brainsci-1176184-supplementary/Table S4.pdf]

Supplementary Table S4 Summary of the statistical results for the slow waves from 700 to 1000 ms (time-locked to S2)

	Time windows		
	700–800 ms	800–900 ms	900–1000 ms
Perturbation [$F_{(2,38)}$]	22.08***	12.54***	6.55**
Front–back [$F_{(2,38)}$]	9.43**	9.66**	6.47**
Left–right [$F_{(2,38)}$]	22.82***	26.96***	24.38***
Perturbation*Front–back [$F_{(2,38)}$]	1.51	0.72	0.74
Perturbation*Left–right [$F_{(2,38)}$]	2.84	0.30	0.72
Front-back*Left–right [$F_{(4,76)}$]	6.13***	5.81**	4.90**
Perturbation*Front−back*Left−right [$F_{(4,76)}$]	0.84	0.80	1.62

Note: * $p < 0.05$; ** $p < 0.01$; *** $p < 0.001$
