# Supplementary material for: Online Movement Correction in Response to the Unexpectedly Perturbed Initial or Final Action Goals: An ERP and sLORETA Study
Source: Brain Sci. 2021 May 15;11(5):641. doi: 10.3390/brainsci11050641 (PMC8156469; doi:10.3390/brainsci11050641)
Supplement: Supplementary file 1 [file brainsci-11-00641-s001.zip › brainsci-1176184-supplementary/Table S5.pdf]

**Supplementary Table S5 Summary of the post hoc results for the factor *perturbation* from 700 to 1000 ms (time-locked to S2)**

| **Contrast** | **Time window** | | |
|---|---|---|---|
| | **700–800 ms** | **800–900 ms** | **900–1000 ms** |
| **FP−IP** | −1.43 | −0.94 | −0.44 |
| **FP−NP** | 4.90*** | 3.79** | 2.89* |
| **IP−NP** | 6.34*** | 4.73*** | 3.33** |

Note: * $p < 0.05$; ** $p < 0.01$; *** $p < 0.001$
