# Supplementary material for: Online Movement Correction in Response to the Unexpectedly Perturbed Initial or Final Action Goals: An ERP and sLORETA Study
Source: Brain Sci. 2021 May 15;11(5):641. doi: 10.3390/brainsci11050641 (PMC8156469; doi:10.3390/brainsci11050641)
Supplement: Supplementary file 1 [file brainsci-11-00641-s001.zip › brainsci-1176184-supplementary/Table S6.pdf]

Supplementary Table S6 Summary of the statistical results for the ERP slow waves from −400 to 0 ms (time-locked to grasping)

	Time windows			
	**−400–−300 ms**	**−300–−200 ms**	**−200–−100 ms**	**−100–0 ms**
Perturbation [$F_{(2,38)}$]	2.09	1.17	1.57	1.99
Front–back [$F_{(2,38)}$]	6.91**	8.29***	9.87***	15.34***
Left–right [$F_{(2,38)}$]	33.14***	36.04***	33.27***	35.54***
Perturbation*Front–back [$F_{(2,38)}$]	0.27	0.69	1.24	0.65
Perturbation*Left–right [$F_{(2,38)}$]	0.92	1.31	1.35	0.73
Front–back*Left–right [$F_{(4,76)}$]	4.14*	3.77*	3.57*	4.48*
Perturbation*Front–back*Left–right [$F_{(4,76)}$]	0.64	0.98	1.00	0.82

Note: * $p < 0.05$; ** $p < 0.01$; *** $p < 0.001$
